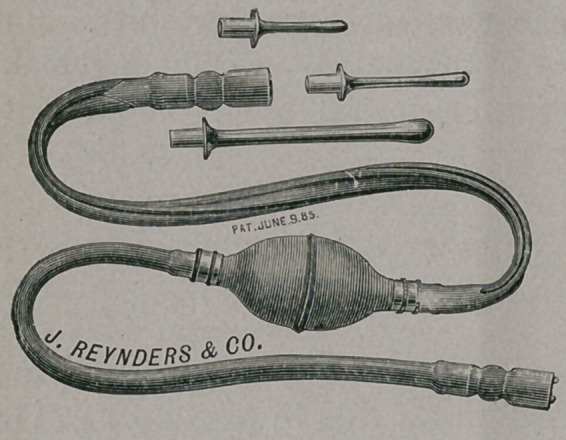# Remedies

**Published:** 1891-07

**Authors:** Herbert Neher


### REMEDIES.

Dr. Herbert Neher, Veterinarian of the Broadway and Seventh Avenue R. R., New York, finds that in treating cases of Influenza "in green stock" (Horses), if there is any tendency to gastric or enteric trouble, "Sour Stomach or purging", the use of the following

℞ Pulv. Carbo Ligni ℥ss.

" Zingib ʒii.

Misce. Ft. Bol. no I

is most successful. He gives three or four in the 24 hours. He likes it better than Tinct. Opii and similar preparations.

---

In Purpura Hæmorrhagica, he injects $\frac{1}{8}$ grain doses of Strychina every four hours and has had excellent results; after the cases are on a fair way to recovery, he gives Iron, Quinine, etc.

He inquires if, after removing the sole or wall of the foot (in horse), any of the readers of THE JOURNAL have tried Dried Sulphate of Iron, dusted on. He finds it forms a crust or scab, excludes the air, has a soothing effect, and is tonic in its action, on the parts.

---

In flatulent colic, when the pelvis is filled by the colon "due to pressure," he punctures through the rectum and saves a wound in the flank. A precaution is necessary however, and that is to keep hold of the canula, for when the gas escapes it recedes with the gut, and if it should slip out of the hand, serious consequences may result.

---

He always gives a full one ounce of aloes, to a horse in full condition, as soon as he enters the Hospital, and uses injections per rectum; and, if the pain is unbearable, etherizes the injection, which seems to have the desired effect.

---

In Spinal Meningitis when the urine has to be drawn with a catheter, he washes the bladder through the catheter, using a rubber syringe with a bulb, like figure, and after the urine is drawn and the bladder washed, he injects a proper amount of Tinct. Nux Vom., which, in coming in direct contact with the mucous surface, seems to tone the parts and they soon regain strength to contract and produce natural micturition.

## Figures and Tables

**Figure f1:**